# Using a Material Library to Understand the Impacts of Raw Material Properties on Ribbon Quality in Roll Compaction

**DOI:** 10.3390/pharmaceutics11120662

**Published:** 2019-12-07

**Authors:** Jiaqi Yu, Bing Xu, Kunfeng Zhang, Chenfeng Shi, Zhiqiang Zhang, Jing Fu, Yanjiang Qiao

**Affiliations:** 1 Department of Chinese Medicine Information Science, Beijing University of Chinese Medicine, Beijing 100029, China; jiaqiyu0126@163.com (J.Y.); zhangkf159@163.com (K.Z.); scf417586179@163.com (C.S.); 2 Beijing Key Laboratory of Chinese Medicine Manufacturing Process Control and Quality Evaluation, Beijing 100029, China; zhangzhiqiang@tcmages.com (Z.Z.); fujing@tcmages.com (J.F.); 3 Beijing Tcmages Pharmceutical Co. LTD, Beijing 101301, China

**Keywords:** roll compaction, material library, roll compaction behavior classification system (RCBCS), design space, ribbon quality, latent variable modeling

## Abstract

The purpose of this study is to use a material library to investigate the effect of raw material properties on ribbon tensile strength (TS) and solid fraction (SF) in the roll compaction (RC) process. A total of 81 pharmaceutical materials, including 53 excipients and 28 natural product powders (NPPs), were characterized by 22 material descriptors and were compacted under five different hydraulic pressures. The transversal and longitudinal splitting behaviors of the ribbons were summarized. The TS-porosity and TS-pressure relationships were used to explain the roll compaction behavior of powdered materials. Through defining the target ribbon quality (i.e., 0.6 ≤ SF ≤ 0.8 and TS ≥ 1 MPa), the roll compaction behavior classification system (RCBCS) was built and 81 materials were classified into three categories. A total of 24 excipients and five NPPs were classified as Category I materials, which fulfilled the target ribbon quality and had less occurrence of transversal splitting. Moreover, the multivariate relationships between raw material descriptors, the hydraulic pressure and ribbon quality attributes were obtained by PLS regression. Four density-related material descriptors and the cohesion index were identified as critical material attributes (CMAs). The multi-objective design space summarizing the feasible material properties and operational region for the RC process were visualized. The RCBCS presented in this paper enables a formulator to perform the initial risk assessment of any new materials, and the data modeling method helps to predict the impact of formulation ingredients on strength and porosity of compacts.

## 1. Introduction

Roll compaction (RC) is a dry agglomeration process, which can be run continuously and has high throughput [1,2,3,4,5,6]. Compared to direct compression (DC), RC is more forgiving of input materials’ bulk density and flowability, and can handle high drug-load formulations [7]. Compared to wet granulation (WG), RC offers the advantages of avoidance of moisture and heat, as well as the decreased production time and operational cost by reducing manufacturing complexity [8,9,10,11]. The RC equipment consists of two counter rotation rollers, which apply high stress to the incoming powder to produce a compacted material, which is called a ribbon or flake [12]. The ribbons are then milled into granules for die compression or encapsulation [13,14,15]. Ribbon properties, such as porosity and strength strongly influence the granules properties like size, fines proportion, flowability and recompactibility [9,16,17,18,19,20], which consequently affect the properties of tablets or capsules.

The ribbon quality control lies in modeling the powder deformation behavior undergoing the RC process. The first choice for predicting ribbon characteristics are one dimensional (1D) models like the Johanson model [21,22,23,24], the slab method [25] and the thin layer model [26]. Johanson’s roll compaction model is the most popular, and it enables the nip angle, the maximum applied normal stress and ribbon relative density to be estimated from inlet material physical properties. However, previous studies have suggested that, in many cases, the Johanson model over-estimates the roll pressure at the nip angle, leading to a larger or non-physical prediction of ribbon relative density [22,27,28]. Besides, 2D and 3D RC process modeling using the finite element method (FEM) can provide more accurate predictions of the ribbon quality attributes than 1D models, since the FEM simulations incorporate multi-dimensional information about the powder state and behavior, feed screw and roll geometry, as well as frictional conditions [29]. Nevertheless, practical application of the FEM models needs large computational effort and a high degree of expert skills. In general, the 1D, 2D and 3D RC models are often limited to the particular material and roller compactor investigated, which restrict them to initial design or mechanism interpretation. The diversity in the powder behavior and the complexity of operating parameters make roller compaction far from being fully controlled.

Understanding how mechanical properties of input material affect ribbon properties is critical to the design and development of RC formulation and process control schemes. A number of researchers have studied the impacts of raw material variation by changing the concentrations and/or types of formulation ingredients (e.g., the active, filler or binder), or using different single materials, on the predictability of ribbon quality attributes [12,30,31,32,33,34]. In a “Quality by Design (QbD)” case carried out by Soh [34], 15 formulations containing a mixture of MCC and lactose were used for ribbon production. The raw material tablet tensile strength, MCC fraction, tapped density, kawakita constant 1/b and angle of fall and span were identified as critical raw material attributes. Pishnamazi [35] designed two different binary formulations containing mainly the microcrystalline cellulose (MCC) 102 with different percentages of lactose and Alcell lignin, respectively. The results illustrated that increasing the lactose or lignin percentage led to higher ribbon density and less limitation in process parameters. Souihi [36] applied seven qualities of dicalcium phosphate (DCP) and thirteen qualities of mannitol to understand the influence of brittle fillers in an ibuprofen tablet formulation on ribbon properties. Each filler was characterized by 10 quality attributes. It was revealed that the material variations did not affect the ribbon thickness nor the amount of fines in the mannitol study, whereas this variation was captured in the DCP model. Mangal [37] investigated the influence of the raw material properties of eight different binders (10%, *w*/*w*) in a paracetamol formulation. A positive correlation was observed between the tensile strength of tablets and the median particle size of the binder. Al-Asady [38] investigated the relationship between the properties of ribbons produced from six single materials, i.e., MCC, hydroxypropyl methylcellulose, maltodextrin, lactose, sodium carbonate and calcium carbonate, with the nano-indentation hardness of their primary particles. It was observed that materials with higher hardness generated ribbons with higher porosity and less tensile strength for all hydraulic pressures. In a recent study, Al-Asady [39] confirmed this conclusion through detailed analysis of eight different materials with two extra materials, i.e., polyvinyl alcohol (PVA) and starch, in addition to the previous six ones. He further noted that particles with low hardness were easily to be bonded together, and fragmentation mechanism did not affect bonding significantly. Seen from the above studies, different authors preferred to use different material characterization tests, and there was a lack of standardized characterization protocol. Besides, the number of materials included in each study was relatively scarce. and there were few studies that indicated what properties of a powder mixture are suitable for the RC unit’s operation.

Recently, the concept of a material library was proposed to obtain sufficient knowledge about raw material properties [40,41]. Building such a material library required systematic characterization of a large set of calibration materials. For instance, Snick [42] created an extensive material property database, in which 55 different raw materials were characterized by over 100 material descriptors. In another study, Benedetti [43] organized a raw material database consisting of 34 active pharmaceutical ingredients (APIs) and seven excipients, and each had eight flowability descriptors. The multivariate linkage of a material library to a specific unit operation has a lot of benefits, including (1) comprehensively and reliably modeling the relationship between raw material properties and process performance; (2) identification of critical raw material attributes and risk assessment of unknown intra- or inter-material variations; and (3) finding tracer material for continuous manufacturing or surrogate materials for high cost APIs. An example was given by Hayashi [44] who built a large dataset containing 81 APIs. The tensile strength of a directly compressed tablet could be well predicted from 12 API characteristics and the compression force. What is more, for example, Wang [45] recognized six clusters of materials from a relatively small material library, and powders from the same group exhibited a similar feeding consistency and accuracy from a twin-screw loss-in-weight feeder. As for roller compaction, Shi first reported the CRAVE database archiving 136 RC formulations [46]. However, only powder tapped densities, roll stress and ribbon solid fraction were recorded. The material library approach has not been systematically investigated for the roller compaction process.

Our work focused on using a material library to understand the impacts of raw material properties on ribbon quality in roll compaction. First, a material library full of material property diversity was built. Each material was characterized comprehensively and was compacted under different hydraulic pressures. The ribbon splitting behavior for different materials were summarized. Then, by characterizing the solid fraction and the tensile strength of the ribbons, a ribbon property database was established. The roll compaction behavior was classified according to the targets set for ribbon properties. Furthermore, the ribbon tensile strength vs. the ribbon porosity curves and the ribbon tensile strength vs. the hydraulic pressure curves were described by the Ryshkewitch–Duckworth and power equations, respectively. Specifically, the latent variable modeling technique was used to explore the multivariate relationships among the raw materials properties, the compaction descriptors, the hydraulic pressures and the ribbon properties. The critical variables affecting the ribbon properties were identified, and a multi-objective design space toward RC formulation and process design was developed.

## 2. Materials and Methods

### 2.1. Materials

A total of 81 pharmaceutical materials, including 53 excipients and 28 natural product powders (NPPs), were chosen to reflect a broad range of physical properties. The detailed information, such as name, lot number and supplier for each material, are given in Table S1 in Supplementary Materials. All excipients were commercially available, and their intended roles in drug formulation were diluents, binders and disintegrants.

Ten kinds of microcrystalline cellulose (MCC), commonly used as plastically deforming materials, were included. The types of these MCC were PH 101, PH 102, PH 301, PH 302, PH 102NF, PH 200NF, Oricel^TM^ PH-102 SCG, Oricel^TM^ PH 302NF, VIVAPUR^®^ type 102 and KG-802. In addition to MCC, cellulose materials such as two batches of methylcellulose (MC), five batches of ethylcellulose (EC), six batches of hydroxypropyl methyl cellulose (HPMC) and one batch of low-substituted hydroxypropyl cellulose (L-HPC) were collected in the material library.

Eight kinds of lactose, which deformed by brittle fracture during compaction, were listed in the material library. The types of lactose were Cellactose^®^ 80, Flowlac^®^ 100, Duralac^®^ H, Anhydrous 21 AN, Granulac^®^ 200, Tablettose^®^ 80, Pharmatose^®^ 110M and Pharmatose^®^ 200M.

Other frequently used fillers in the material library were mannitol, corn starch, pregelatinized starch, dextrin, sucrose, glucose, sorbitol (two batches) and calcium phosphate dibasic (DCP, two batches). Included also were various disintegrating agents, such as carboxymethyl starch sodium (CMS-Na, two batches), croscarmellose sodium (CCNa, three batches), polyplasdone (PVPP, two types) and calcium carboxymethylcellulose (CMC-Ca). Besides, the material library included calcium phosphate, sodium bicarbonate and DL-malic acid.

All NPP materials were provided by the Beijing Tcmages Pharmaceutical Co., Ltd. (Beijing, China) and were manufactured under GMP (Good Manufacturing practice) certified environments. The manufacturing process consisted of a series of unit operations, such as preprocessing of crude drugs, water extraction, filtration, vacuum concentration and drying. In terms of drying methods, 11 batches of NPPs (No. 54 to No. 64) were prepared by spray drying, and 17 batches of NPPs (No. 65 to No. 81) were prepared by vacuum belt drying. Different production batches for the same material were also considered. NPPs were proved to possess high hygroscopicity, viscosity and poor flowability [47,48]. In this paper, 28 batches of NPPs prepared from 12 plant materials were expected to expand the variation coverage of physical properties in the material library.

### 2.2. Powder Characterization

Each powdered material was characterized by 22 parameters that reflected different aspects of material properties, such as particle size, density, flowability, compressibility, stability and texture. Among them, 12 material descriptors, namely bulk density (*Da*), tapped density (*Dc*), inter-particle porosity (*Ie*), cohesion index (*Icd*), Carr index (*IC*), Hausner ratio (*IH*), angle of repose (*AOR*), flow time(*t″*), moisture content (*%HR*), hygroscopicity (*%H*), percentage of particle size smaller than 50 μm (*%Pf*) and homogeneity index (*Iθ*), were measured or calculated according to standard test procedures of the SeDeM expert system methodology [49].

The bulk density (*D_a_*, g/mL) was measured by gently filling powders (*m* in g) into a 250 mL graduated cylinder. The volume of powders was recorded as *V_a_* in mL. The tapped density (*D_c_*, g/mL) was determined by a tap density tester (HY-100; Dandong Hengyu Instrument Co., Ltd., Dandong, China). The cylinder filled with powder was fixed on the tester, and the number of vibrations was 1250 times. The resulting volume (*V_c_* in mL) was recorded. The bulk density and the tapped density were calculated using Equations (1) and (2):

(1) $$D_{a\;}\;=\;\frac{m}{V_{a}},$$

(2) $$D_{c}\;=\;\frac{m}{V_{c}}.$$

The inter-particle porosity, the Carr index and the Hausner ratio can be computed according to Equations (3)–(5), respectively:

(3) $$Ie\;=\;\frac{D_{c}\;-\;D_{a}}{D_{c}\;\times \;D_{a}},$$

(4) $$IC\;=\;\frac{D_{c}\;-\;D_{a}}{D_{c}}\;\times \;100,$$

(5) $$IH\;=\;\frac{D_{c}}{D_{a}}.$$

The cohesion index (*Icd*) is denoted by the hardness of the powder being compressed into the tablet. About 350 mg powder was compacted into tablet on a single punch press (ZYP-30TS, Shanghai Xinnuo Instrument Co., Ltd., Shanghai, China). The diameter of the flat face punch was 10 mm, and the compression force was 1 ton. Magnesium stearate was used to lubricate the upper and lower punches face and die walls before tableting. The hardness (N) of the tablet was obtained by a crushing force tester (YPD-500C, Shanghai Huanghai Drug Testing Instrument Co., Ltd., Shanghai, China).

The angle of repose (AOR) and flow time (*t″*) were characterized by the powder fluidity tester (BEP2, Copley Instrument Co., Ltd., Nottingham, Britain). About 100 g powder were weighted and were poured slowly onto a fixed round platform through a funnel until a stable powder cone was formed. The height (*h*) and the diameter (*r*) of the powder cone were measured. AOR was calculated through Equation (6). The time required for the same amount of powder to flow out from the funnel was recorded as *t″*:

(6) $$\tan\left(AOR\right)\;=\;\frac{2h}{r}.$$

The moisture content (*%HR*) was characterized by the Sartorius MA35 instrument (Sartorius AG, Goettingen, Germany). Approximately 2.0 g of powder was evenly spread onto the sample tray and was heated at 105 °C for 10 min. The difference between weights of a powder sample before and after it is dried was taken as the percentage of moisture content.

The hygroscopicity (*%H*) was tested as follows. A dry bottle was weighed and placed in a constant temperature drier at 25 ± 1 °C. After 12 h, the weight of the bottle (*m*_1_) was measured. Then, the powder sample (about 1 mm in height) was tiled into the bottle and their weight (*m*_2_) was measured. The bottle was opened and placed in a drier with the relative humidity of 80% (±2%) of and the temperature of 25 °C (±1 °C). After 24 h, the weight of bottle with powder (*m*_3_) was (*m*_3_). *%H* was calculated by Equation (7):

(7) $$\%H\;=\;\frac{m_{3}-m_{2}}{m_{2}-m_{1}}\;\times \;100\%.$$

The true density (*D_t_*) of powder was determined by the pore size analysis instrument (3H-2000PS1, Beishide Instrument technology Co., Ltd., Beijing, China). Firstly, the sample cell and filling rod are installed, and the true density test program is selected to test the volume *V*_1_ of the empty cell. The weight (*m*) of the sample is measured and is added into the empty cell, and the amount of sample added should not exceed 2/3 of the volume of the cell. *V*_2_ is the volume after loading the sample. When the coefficient of variation of the three measurements was below 0.05%, the experiment was terminated and *D_t_* calculated by Equation (8). The mean of the three measurement was reported as *D_t_* of the sample.

(8) $$D_{t}=\frac{m}{V_{1}-V_{2}}.$$

Once *D_t_* was obtained, the powder solid fraction (*SF_p_*) and powder porosity (*ε_p_*) could be calculated according to Equations (9) and (10), respectively.

(9) $$SF_{p}\;=\;\frac{D_{a}}{D_{t}},$$

(10) $$\varepsilon_{p}\;=\;1-SF_{P}.$$

The particle size distribution (PSD) was determined by the laser diffraction particle size analyzer (BT-2001, Dandong Baite Instrument Co., Ltd., Dandong, China) with the dry powder module. Background calibration is performed before testing. About 3 g of powder sample is placed on the vibrational feeder unit. The powder sample is inhaled by vacuum to pass through a laser beam. The air pressure is maintained at 0.2 MPa to make the dispersion of powder effective. The shading rate is adjusted in the range of 0.5–5% according to properties of the powder. The measurement is repeated three times for each material and the mean particle size distribution was calculated. *D*_10_, *D*_50_ and *D*_90_ values are the particle size values at which 10%, 50% and 90% of the particles fall below. Span represents the width of particle size distribution and is defined as Equation (11).

(11) $$span\;=\;\frac{D_{90}-D_{10}}{D_{50}}.$$

The percentage of particle sizes smaller than 50 μm (*%Pf*) was estimated from the PSD. The homogeneity index (*Iθ*) was determined by Equation (12):

(12) $$I\theta \;=\;\frac{F_{m}}{\begin{array}{c} 100+\left(d_{m}-d_{m-1}\right)F_{m\;-\;1}+\left(d_{m+1}-d_{m}\right)F_{m+1}+\left(d_{m}-d_{m-2}\right)F_{m-2}\\ +\;\left(d_{m+2}-d_{m}\right)F_{m+2}\cdots +\left(d_{m+n}\;-\;d_{m}\right)F_{m+n} \end{array}}$$

where *F_m_* is the percentage of powder in the majority range; *F_m_*_+1_ and *F_m−_*_1_ are the percentages of powders in the range immediately above and below the majority range; *n* is the order of the fraction studied relative to the series of majority fraction; *d_m_* is the mean diameter of the powder in the majority fraction; and *d_m_*_+1_ and *d_m_*_−1_ are the mean diameter of the powder in the fraction of the range immediately above and below the major range.

The springiness, cohesiveness and springiness coefficient were characterized by a texture analyzer (TMS-Touch, Food Technology Corporation Co., Ltd., Sterling, VA, USA) with TPA mode. The instrument is equipped with a 1000 N load cell and a cylindrical probe with a 38.11 mm diameter. The powder sample is loosely piled in an iron sample tray into the height of about 2 mm. The iron sample tray has a diameter of 52 mm and height of 15 mm. The deformation rate is set at 30% of the volume of the sample. The test speed is maintained at 30 mm/min and the trigger force is set to 1.5 N.

The powder morphology was examined using scanning electron microscopy (SEM, JSM-7001F+INCA X-MAX, Tokyo, Japan). First, about 15 conductive carbon tapes, each of which had the dimension of 5 mm× 5 mm, were attached to the aluminum sample board. Then, a small amount of powder samples was glued to the conductive carbon tapes. After that, the powder samples were gold coated using an Auto Fine Coater (JEC-3000FC, JEOL Ltd., Tokyo, Japan) and the coating time was 120 s. The coated samples were tested at a current of 20 mA and voltage of 5–10 kV. The morphology of the particles was observed at different magnifications.

Each powder was forced to pass through an 850 μm ± 29 μm aperture size sieve to remove any lumps present before testing. The powder characterization data were archived into the homemade iTCM database [50], in which the properties of 53 excipients could be accessed from the website (http://info.pharm.bucm.edu.cn/xsgz/sjgxpt/48350.htm). The measured physical properties of 28 NPPs are shown in Table S1.

### 2.3. Roll Compaction and Ribbon Production

The powders were compacted into ribbons using the LGS120 roller compactor (Beijing Longli Tech Co., Ltd., Beijing, China). The roller compactor was equipped with a feeding system that included a hopper and a horizontal screw feeder, which conveyed the powder to the vertical positioned rollers. The diameter and width of the knurled surface rollers were 120 and 35 mm, respectively. Two cheek plates were placed on both sides of the rolls to prevent the powder from leaking during roll compaction. One fixed and one floating roll configuration was applied to maintain the hydraulic pressure to be constant. The values of pressure in bar were continuously measured by a calibrated pressure gauge and were displayed on the control panel, but the roll force was not directly measured. The gap size between the rollers could vary depending on the state of fed material.

In the literature [34,35,51,52,53,54], it was demonstrated that the roll pressure was the most important process parameter that affected the resulting ribbons. In this paper, only the hydraulic pressure was considered as the critical process parameter (CPP) in order to fully understand the impacts of raw material properties on ribbon qualities. Other process conditions were kept as constant as possible. For all materials, different hydraulic pressures in a range of 30–110 bar and at 20 bar increments were used to compact the powder into ribbons. The roll speed and the screw speed were adjusted to 6 rpm and 20 rpm, respectively. Prior to compaction, a vacuum de-aeration system was switched on and took the air away from the powder. The magnesium stearate was used to lubricate each roller surface using a soft brush. A cooling system was employed, and the temperature of the liquid circulating inside the roller was maintained around 14 °C. The experiments were carried out under room temperature held between 18 °C and 22 °C and the relative humidity varying between 40% and 50%.

About a 3 kg batch for each material was prepared. Once the roller compactor was started, at least 1 min was lasted to assure that the steady-state of ribbon formation was achieved. The milling system was stopped in order to produce ribbons with intact shape and sufficient lengths. The collected ribbons were carefully de-dusted using a soft brush. Before ribbon characterization, the ribbon samples were stored for at least 24 h.

### 2.4. Ribbon Characterization

#### 2.4.1. Ribbon Solid Fraction

The ribbon solid fraction (SF) or ribbon relative density is calculated using the following equation:

(13) SF = 1 − ε.

It is based on the measurement of ribbon porosity (*ε*), which is defined as void fraction in a compacted material. The overall ribbon porosity was determined by the oil intrusion method [55,56]. The obtained ribbon was first cut into sample pieces with the length of 30 to 50 mm. Then, each sample ribbon was weighed and immersed in liquid paraffin oil (Ch. P. grade, Shangqiu Liangfeng Health Products Co., Ltd., Shangqiu, China) carried in a Petri dish. The Petri dish was kept under vacuum (approximately 10 Pa) for 15 min. Once the vacuum was released, the sample ribbons were allowed to absorb oil into their internal pores for 30 min. The oil-saturated ribbons were prepared by carefully wiping off the superficial oil with the filter paper. After that, the weight of oil absorbed by the oil-saturated ribbon is used to estimate the porosity of the ribbon by Equations (14)–(16) as follows:

(14) $$V\left(oil\right)\;=\frac{m\left(oil\;+\;ribbon\right)\;-\;m\left(ribbon\right)}{\rho \left(oil\right)},$$

(15) $$V\left(ribbon\right)\;=\;\frac{m\left(ribbon\right)}{\rho \left(powder_{true}\right)},$$

(16) $$\varepsilon \;=\;\frac{V\left(oil\right)}{V\left(oil\right)\;+\;V\left(ribbon\right)}\;\times \;100,$$

where ρ(oil) represents the density of paraffin oil; V(oil) represents the volume of paraffin oil; m(oil+ribbon) represents the weight of the oil-saturated ribbon; m(ribbon) represents the weight of the original ribbon; V(ribbon) represents the solid volume in the ribbon; and $\rho \left(powder_{true}\right)$ represents the true density of the material. Since no mixture was involved in this paper, $\rho \left(powder_{true}\right)$ was equivalent to *D_t_* of the raw material. Reported results were the mean value of three repeated experiments.

#### 2.4.2. Ribbon Tensile Strength

The ribbon tensile strength (TS) was quantified using the three-point bending method [57,58,59]. Before testing, samples were prepared by cutting ribbons into the approximate lengths of 20 mm. The width and thickness of the ribbons were measured using a digital caliper (SL01-22, Shanghai DingLeng Industrial Development Co., Ltd., Shanghai, China). The analysis was performed on a Texture Analyzer (TMS-Touch, Food Technology Corporation Co., Ltd., Sterling, VA, USA) with a 50 N load cell. The ribbon sample was placed on two supports whose distance between each other was 15 mm. A load was then applied to the center of the ribbon at a constant speed of 12 mm/min. The force increased gradually until the ribbon broke. The ribbon tensile strength was calculated according to Equation (17):

(17) $$TS\;=\;\frac{3FL}{2WT^{2}}$$

where *F* (N) represents the force at breakage; *L* (mm) is the distance between two lower supports; *W* (mm) is the sample width; and *T* (mm) is the sample thickness. For each ribbon sample, repeated measurements were made at least three times to obtain the average *TS*.

#### 2.4.3. Ribbon Morphology

The ribbon samples were broken gently. The small pieces of ribbons (about 3–5 mm^2^) were attached to conductive carbon tapes on the aluminum sample board. Then, a conductive carbon tape with about 1 mm × 10 mm dimensions was fixed on each ribbon sample to prevent it from falling during the test. Other analyzing procedures were consistent with the SEM method illustrated in Section 2.2.

### 2.5. Empirical Model Fitting for Roll Compaction

The macroscopic TS and SF of ribbons produced by RC were similar to that of slugs produced by die compaction [60]. Therefore, the strength-porosity model and the strength-pressure model are chosen to explain the roll compaction behavior of powdered materials.

#### 2.5.1. The Ryshkewitch–Duckworth Model

The Ryshkewitch–Duckworth (R–D) equation described an exponential relationship between tensile strength and porosity of the compact [61]:

(18) $$Ln\left(\frac{TS}{TS_{0}}\right)\;=\;-k_{b}\varepsilon$$

where *TS*_0_ is the intercept with the *y*-axis, the physical meaning of which is the tensile strength of the compact at zero porosity. The slope *k_b_* represents the bonding capacity between particles. A larger value of *k_b_* implies weaker bonding of primary particles. While, an increase in the value of *TS*_0_ indicates the material becomes less deformable [62]. The R–D equation was demonstrated to be suitable for a wide range of powdered materials [63,64,65,66,67,68]. Several researchers have revealed that this strength–porosity model fitting could be used to explain exponential increase in ribbon *TS* with rising ribbon SF [30,58,69].

#### 2.5.2. The Power Model

Osborne [70] and Omar [12] observed that the strength of the ribbon was dependent mainly on the hydraulic pressure. In this study, the relationship between the ribbon TS and the hydraulic pressure is described by a simple power equation as follows:

(19) $$TS\;=\;dP^{g}$$

where *P* is the hydraulic pressure, and *d* and *g* are fitted constants.

### 2.6. Multivariate Analysis

The partial least squares (PLS) regression method was used to build a model for the input data matrix and the output data matrix [71,72]. Performing a PLS analysis can help reduce the input dataset to a few latent variables that are linear combinations of the original variables. The correlations between the variables could be better understood. The similarities and dissimilarities between observations could be easily assessed. In this work, the PLS regression was carried out using SIMCA 13.0 (Umetrics, Umea, Sweden) software.

## 3. Results and Discussion

### 3.1. The Powder Properties

**Figure 1:**
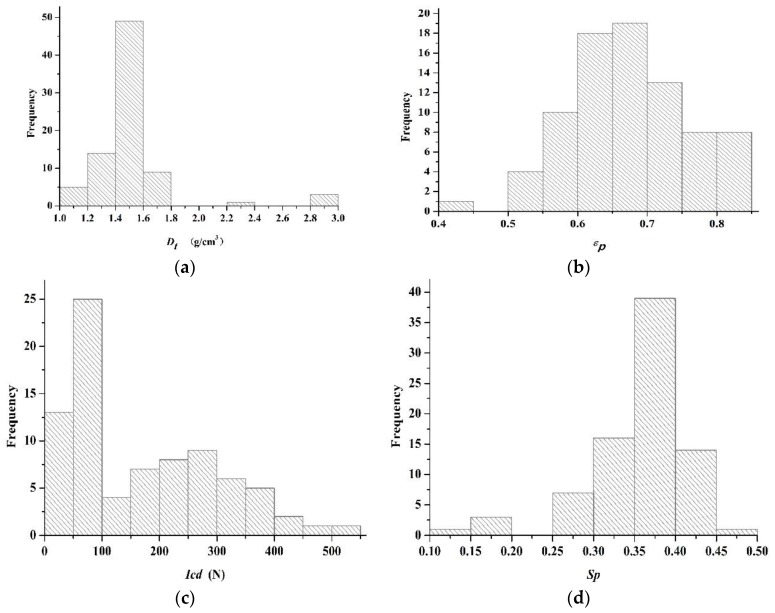
The frequency distribution histograms of four representative powder properties. (**a**) The true density (*D_t_*); (**b**) the powder porosity (*ε_p_*); (**c**) the cohesion index (*Icd*); and (**d**) the springiness.

As a result of comprehensive powder characterization, 22 physical properties of 81 powders are shown in Table S1 in the Supplementary Materials. Four representative properties, i.e., the true density, the powder porosity, the cohesion index and the springiness, were chosen for brief analysis, and their frequency distribution plots are shown in Figure 1. As seen in Figure 1a, the true densities of most materials are spread in the range between 1.145 g/cm^3^ (i.e., EC, No. 15) and 1.634 g/cm^3^ (i.e., MCC, No. 10). Besides, DCP (No. 41), DCP (No. 42), Ca_3_(PO_4_)_2_ (No. 51) and NaHCO_3_ (No. 52) had relatively large true densities that are 2.915 g/cm^3^, 2.862 g/cm^3^, 2.818 g/cm^3^ and 2.230 g/cm^3^, respectively. The range of powder porosity (*ε_p_*) is wide, as shown in Figure 1b, beginning from 0.4479 (sucrose, No. 37) up to 0.8456 (MCC, No. 10). The cohesion index (*Icd*) indicates whether the powder can form proper bonds and a large *Icd* value is linked with adequate compactability [73]. The *Icd* values for all materials vary between 0 N (i.e., d-(+)-glucose, No. 38) and 503.0 N (i.e., MC, No. 12). The D-(+)-glucose could not be compacted into tablet, so its *Icd* is recorded as zero. The springiness values of powders vary from 0.1255 (i.e., lactose Pharmatose^®^ 200 M, No. 32) to 0.4600 (i.e., sodium bicarbonate, No. 52). From the above analysis, it can be seen that there are notable differences between powders, which is beneficial for increasing the diversity of the material library.

### 3.2. The Ribbon Characteristics

According to Section 2.3, all 81 materials in the material library were compacted under hydraulic pressures of 30, 50, 70, 90 and 110 bar, respectively. Since the magnesium stearate as the lubricant was brushed onto each roller surface continuously, all materials were not sticking to the rollers. The ribbon characteristics are described from both the macro and micro points of view.

In theory, 405 (i.e., 81 × 5) ribbon samples or ribbon-like compacts could be prepared. However, two powders could not be compacted at low hydraulic pressures. The CMS-Na (No. 44) and CCNa (No. 46) were found to hold the powder state after being compacted under 30 bar and 50 bar. This suggested that the relatively low pressures applied are not enough to plastically deform the two materials or to cause their particles to bond together. Besides, at low hydraulic pressures, it is observed that ribbons produced from different materials had different widths. For instance, cellulose materials generated ribbons with 35 mm in width, which is the same as the roller width. It indicates that the low hydraulic pressure applied is sufficient to squeeze and bond the plastic particles together. By contrast, the same low pressure is insufficient to bond the brittle material to produce full width ribbons. For example, the Lac-A (No. 28) generates ribbons with the average width of 30 mm at 30 bar. While, the compacts of DCP (No. 41) have the width of 28 mm at 30 bar.

**Figure 2:**
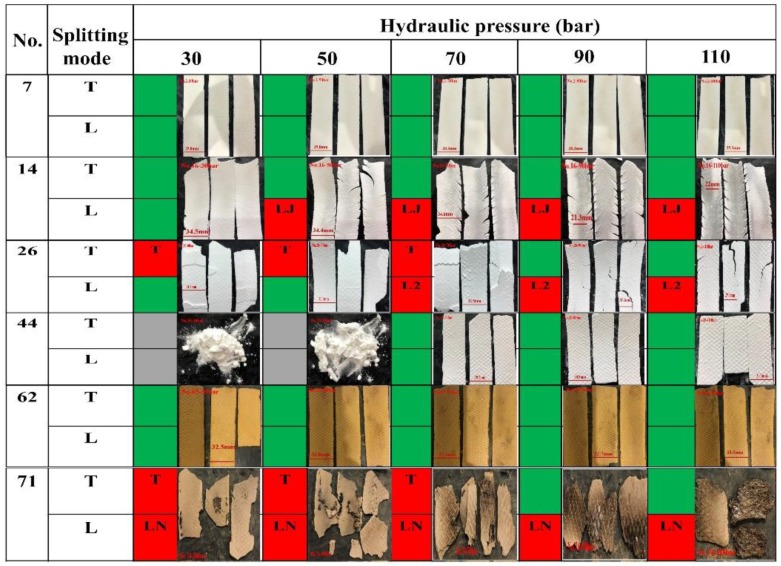
Effect of different hydraulic pressures on the splitting behavior of ribbons. Splitting is designated by “T” (transversal splitting), Ln (longitudinal splitting into n pieces) and “LJ” (longitudinally joined splitting). Gray region represents the powder cannot be compacted into ribbons. Red region indicates the occurrence of splitting “T” or “L”. Green region corresponds to conditions under which splitting “T” or “L” does not occur. No. 7 is MCC Oricel^TM^ PH-102 SCG, No. 14 is Ethyecellulose N-10 Pharm, No. 26 is lactose Flowlac^®^ 100, No. 44 is Carboxy methyl starch sodium, No. 62 is Polygoni Multiflori Radix Praeparata extracts and No. 71 is Rehmanniae Radix extracts.

Ribbon splitting may occur transversally (through the ribbon thickness) or longitudinally (through the ribbon width) [74]. Table S1 was constructed to better display the splitting characteristics of ribbons produced under different pressures. The transversal and longitudinal splitting are termed as “T” and “L”, respectively. In Table S1, data cells with background color in red represent the occurrence of the splitting “T” or “L”. While, data cells with background color in green represent that intact ribbons are obtained. Gray data cells mean ribbons do not form as discussed above. Since Table S1 is large, the split modes of six representative materials, i.e., MCC 102 (No. 7), EC (No. 14), Lac-F (No. 26), CMS-Na (No. 44), Polygoni Multiflori Radix Praeparata extracts (No. 62) and Rehmanniae Radix extracts (No. 71), are taken out to form a new table together with ribbon pictures, as shown in Figure 2.

For MCC102 (No. 7) and Polygoni Multiflori Radix Praeparata extracts (No. 62), intact ribbons are obtained under all hydraulic pressures and there is no splitting. The EC (No. 14) powder form intact ribbons at the hydraulic pressure of 30 bar. However, the longitudinal splitting from the edge occurs at the hydraulic pressure from 50 bar to 110 bar, and the cracks are joined. This particular splitting mode is named as “LJ”. The reason may be attributed to the oscillatory powder feeding and the friction of particles with the wall, which lead to non-uniform roll pressure and resulted ribbons across the ribbon width and along the rolling direction [26,70,75,76,77]. For ribbons of Lac-F (No. 26), the splitting “T” occurs at 30, 50 and 70 bar, while the splitting L2 (i.e., the ribbon is divided into two sections through-width) occurs at 70, 90 and 110 bar. Some powders (e.g., Rehmanniae Radix extracts, No. 71) form flakes that do not possess sufficient mechanical strength and crumble into small fragments upon pressing. This splitting mode is named as “LN”.

For all materials compacted, the frequency of splitting occurrences under different hydraulic pressures are summarized in Table 1. The frequencies of transversal splitting are 39, 40, 34, 31 and 26 under 30, 50, 70, 90 and 110 bars, respectively. The frequencies of splitting for L2, L3, LJ and LN splitting modes were counted separately. And the total frequencies of longitudinal splitting are 10, 26, 43, 48 and 49 under 30, 50, 70, 90 and 110 bars, respectively. Obviously, there is a trend that the frequencies of transversal splitting decrease as the hydraulic pressure is increased. By contrast, the frequencies of longitudinal splitting increase as the hydraulic pressure is increased. Plastic materials have more occurrence of “L” splitting and brittle materials are more likely to show “T” splitting, which is related to the structure and properties of the particles themselves [78,79,80].

**Figure 3:**
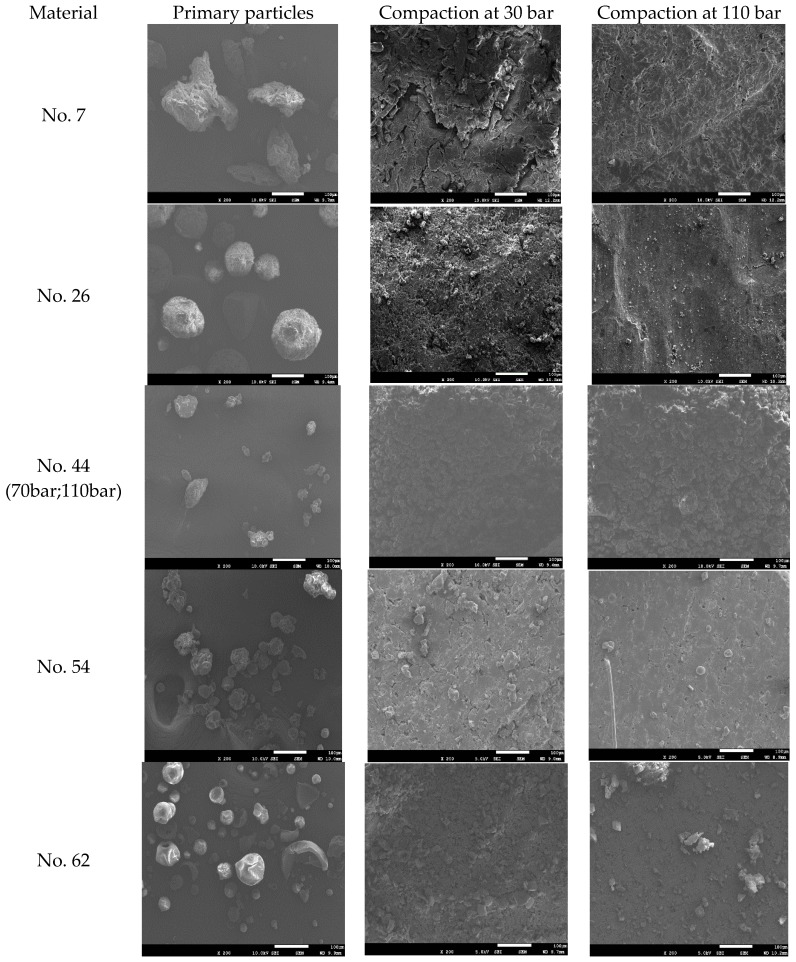
SEM images of primary particles and ribbon surfaces of five typical materials produced at different hydraulic pressures. No. 7 is MCC OricelTM PH-102 SCG, No. 26 is lactose Flowlac^®^ 100, No. 44 is Carboxy methyl starch sodium, No. 54 is Mume fructus extracts and No. 62 is Polygoni Multiflori Radix Praeparata extracts.

Figure 3 shows the SEM images of the primary particles and the corresponding ribbon surfaces of five typical materials. The ribbon surface morphology of MCC 102 (No. 7) produced under the hydraulic pressures of 30 and 110 bar shows significant plastic deformation, resulting in a change in the shape of particles. As the hydraulic pressure increases, the deformation increases, which may contribute to the high rate of increase in ribbon strength as the compaction pressure increases [38]. At the pressures of 30 or 110 bar, the ribbon surface of the Lac-F (No. 26) still has several smaller particles, which are signs of breakage. The fracture and insignificant plastic deformation of the lactose can help explain why no strong ribbon and plastic deformation are produced [12]. The SEM images of CMS-Na (No. 44) are available at pressures of 70 bar and 110 bar. As the pressure increases, the particles hardly undergo a deeper deformation. The ribbon surface of CMS-Na is rough, and the shape of the primary particles could be seen clearly. Due to the limited amount of breakage and deformation, the produced ribbons of CMS-Na are porous and are expected to be weaker than MCC and lactose [12]. The SEM images of two NPPs, i.e., Mume Fructus extracts (No. 54) and Polygoni Multiflori Radix Praeparata extracts (No. 62), show that their primary particles approximate the spherical shape, and the surface of the No. 54 particle is rough. At 30 bar pressure, there are many particles on the surface of the ribbon, and the voids between particles are large. At 110 bar pressure, both the particles on the ribbon surface and pore spaces between particles are significantly reduced. For ribbons produced under high hydraulic pressures, the micro-sintering or sintering of particles can be clearly observed for some materials, such as cellulose (e.g., MCC 102, No. 7), lactose (e.g., Lac-F, No. 26) and NPPs (e.g., Mume Fructus extracts, No. 54).

### 3.3. Description of Roll Compaction Behavior

#### 3.3.1. Relationship between Ribbon Tensile Strength and Porosity

The Ryshkewitch–Duckworth equation is used to predict the variation of ribbon tensile strength with the ribbon porosity for various single materials in the material library. By means of the least square algorithm, values of two coefficients of the R–D equation are estimated and help explain how well a powdered material can be compacted. The goodness of fit is evaluated by the determinant coefficient R^2^. The fitting results for each material are given in Table S1. The ribbon tensile strength of two NPPs (No. 71 and No. 72) cannot be tested since the obtained ribbons cannot be cut into desirable shapes. Therefore, the model fitting for the two materials is not possible. Besides, the TS-porosity curves of 15 materials, including 11 excipients and four NPPs, do not change monotonically and cannot be fitted well (R^2^ < 0.7). For another two materials, i.e., d-(+)-glucose (No. 38) and DCP (No. 41), the estimated coefficients are unreasonable. For example, the value of *TS*_0_ estimated for DCP (No. 41) is 273,500 MPa, and this may be explained by the limited porosity changes (i.e., 0.311–0.338) under the pressure range applied. These results demonstrated that the R–D equation is not applicable in all situations [81].

**Figure 4:**
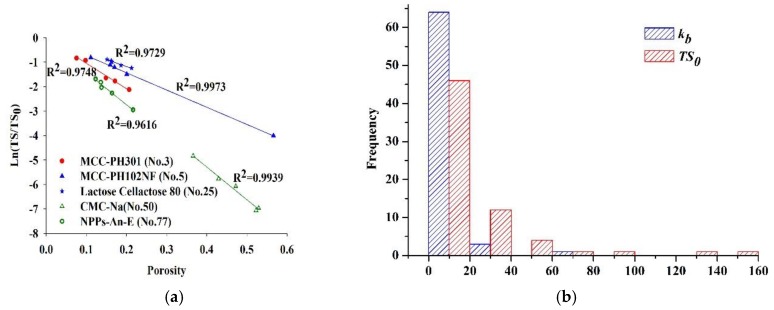
(**a**) The linear relationship between *Ln*(*TS*/*TS*_0_) and ribbon porosity for five typical materials. (**b**) The frequency distribution of fitted parameters *k_b_* and *TS_0_* in the Ryshkewitch–Duckworth equation.

The R^2^ values of the remaining 62 batches of materials are spread in the range from 0.7388 to 0.9973, demonstrating the good or fair fit quality. The linear relationship between *Ln*(*TS*/*TS*_0_) and ribbon porosity for five typical materials, i.e., MCC 301 (No. 3), MCC 102NF (No. 5), Lac-C (No. 25), CMC-Ca (No. 50) and *Anemarrhena asphodeloides* Bunge extracts (No. 77), are shown in Figure 4a. Although the pressure range used is equivalent, either the porosity or TS ranges varies due to the different roll compaction behavior of each material. Ribbons made from MCC 102NF (No. 5) have a wide range of porosity distribution (i.e., 0.43–0.89) as well as a wide range of tensile strength distribution (i.e., 0.44–10.85 MPa). By contrast, ribbons made from Lac-C (No. 25) have narrow ranges of porosity distribution (i.e., 0.79–0.85) and tensile strength distribution (i.e., 2.37–3.36 MPa). At the same porosities, MCC 102NF (No. 5) ribbons are stronger than MCC 301 (No. 3) ribbons. Within the hydraulic pressure range applied, the high porous structure of ribbons of CMC-Ca (No. 50) allows this material to act as agent for disintegration of tablet.

Figure 4b shows frequency distributions of successfully fitted parameters *k_b_* and *TS*_0_ for 62 batches of materials. The compaction descriptor *TS*_0_ indicates the inherent bonding propensity. In this paper, the *TS*_0_ values are in the range from 0.8457 MPa (Lac-P, No. 32) to 144.8 MPa (CMC-Ca, No. 50), and are concentrated between 0 and 40 MPa. Since *TS*_0_ is extrapolated at zero porosity, the value of *TS*_0_ is not unexpected to be higher than the maximum tensile strengths measured. *k_b_* is related to plasticity and the inherent cohesiveness of a powder [82]. The *k_b_* values vary between 2.954 (Lac-P, No. 32) and 32.28 (sodium bicarbonate, No. 52) and are mainly distributed between 0 and 20. Under that same of level of *TS*_0_, the higher value of *k_b_* indicates that the ribbon TS increases faster as porosity is reduced. The *TS*_0_ values of Lac-C (No. 25) and Anemarrhenae rhizome extracts (No. 77) are 8.122 MPa and 8.353 MPa, respectively. The *k_b_* value of Anemarrhenae rhizome extracts (No. 77) is 13.85, which is greater than that of Lac-C (*k_b_* = 5.90). At the same porosity, e.g., 0.836, the *TS* of Lac-C ribbons (i.e., 3.01 MPa) is higher than that of Anemarrhenae rhizome ribbons (i.e., 0.87 MPa). These results indicate that higher value of *k_b_* corresponds to the higher interparticle bonding capacity, but it does not determine the magnitude of ribbon strength.

#### 3.3.2. Relationship between Ribbon Tensile Strength and Hydraulic Pressure

**Figure 5:**
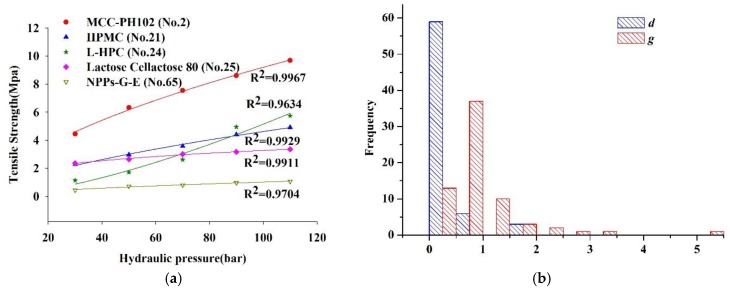
(**a**) The nonlinear relationship between tensile strength and hydraulic pressure for five typical materials. (**b**) The frequency distribution of fitted parameters *d* and *g* in the power equation.

The dependence of ribbon tensile strength on hydraulic pressure (HP) is described by a simple power equation according to Equation (19). The TS–HP curve is fitted by the Levenberg–Marquardt algorithm, and the coefficients *d* and *g* of the power equation are obtained. The fitting results are given in Table S1. It is noted that the power equation is not applicable to 16 materials (R^2^ < 0.7), whose TS–HP profiles do not change monotonically. The TS of DCP (No. 41) increases from 0.71 MPa at the HP of 90 bar to 1.51 MPa at the HP of 90 bar, and this sudden curve change cannot be explained well by the power equation (R^2^ = 0.6339). The curve fitting for two NPPs (No. 71 and No. 72) is also not performed as discussed above. As a result, the R^2^ values of the remaining 62 materials, including 41 excipients and 21 NPPs, are larger than 0.7. The nonlinear relationship between ribbon TS and HP for five typical materials, i.e., MCC 102 (No. 2), HPMC (No. 21), L-HPC (No. 24), Lac-C (No. 25) and Glycyrrhizae extracts (No. 65), are shown in Figure 5a. The frequency distributions of the fitted parameters *d* and *g* for the 62 materials are shown in Figure 5b.

The *g* values range from 0.2771 (Lac-C, No. 25) to 5.20 (D-glucose, No. 38). The compaction descriptor *g* denotes the pressure sensitivity of the material and determines the shape of the compaction profile. When the *g* value is larger than 1, the concave compaction profile (e.g., L-HPC, No. 24) can be observed. On the contrary, the convex compaction profile (e.g., MCC PH102, No. 2) is obtained when the *g* value is smaller than 1. A straight line (e.g., Lac-P1, No. 31) represents that the *g* value is equal to 1. The *d* values range from 5.79 × 10^−11^ (d-(+)glucose, No. 38) to 1.86 (MCC KG802, No. 10). It is found that the *d* values are moderately correlated with the TS of ribbons compacted at the HP of 30 bar (R^2^ = 0.6796). This indicates that the compaction descriptor *d* contributes to the initial ribbonability of the powdered materials. For instance, the *g* values of HPMC (No. 21) and G-E (No. 65) are 0.6194 and 0.6083, respectively, but their *d* values are 0.2667 and 0.0619, respectively. This reveals that the compaction profiles of the two materials are almost the same, but the ribbon TS of HPMC (No. 21) is stronger than that of G-E (No. 65). Besides, most of cellulosic materials (e.g., MCC PH102, No. 2) have large *d* values, and these materials could be easily roller compacted into ribbons with high tensile strength even under low hydraulic pressures. The L-HPC (No. 24) has a low *d* value (i.e., 5.76 × 10^−3^) and high *g* value (i.e., 1.47), and it can be compacted into ribbons with large TS at relatively high hydraulic pressures. The Lac-C (No. 25) has a high *d* value (i.e., 0.9136) but low *g* value, implying it is not sensitive to changes in hydraulic pressures.

### 3.4. Roll Compaction Behavior Classification System

As intrinsic ribbon properties, solid fraction and tensile strength play a major role in the compressibility of downstream granules during tableting. Considerations should be given to the targets of ribbon properties. As far as we know, Hancock et al. [83] first proposed that the solid fractions of the roller compacted ribbons ranged from 0.6 to 0.8, on the basis of 15 roller compacted formulations and excipients. This typical range of ribbon solid fraction (i.e., 0.6–0.8) was further confirmed for a range of excipients and formulations used for immediate and controlled release tablets [30,57]. Compared to the generally accepted solid fraction range for commercial tablet (i.e., 0.8–0.9) [84], a distribution of 0.6–0.8 for ribbon seems reasonable, since lower ribbon solid fraction would result in lower loss of tabletability [17,85]. When the ribbon solid fraction was larger than 0.8, a significantly reduced tabletability would be observed [85]. Besides, it was suggested by the Manufacturing Classification System (MCS) working group [84] that the ribbon tensile strength should be above 1 MPa to ensure adequate mechanical strength for milling. Therefore, a solid fraction of 0.6–0.8 together with a tensile strength larger than 1 MPa are used as the evaluation target to distinguish the roll compaction behavior of different materials.

**Figure 6:**
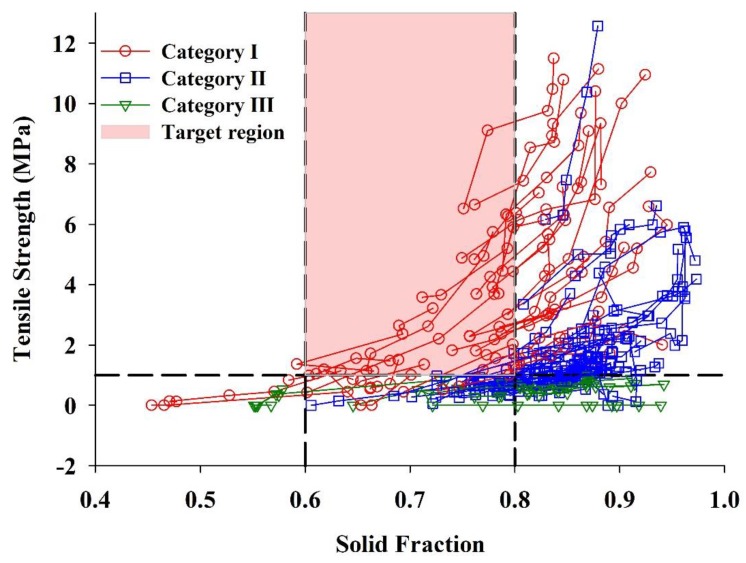
A roll compaction behavior classification system for investigated materials in the material library (each point represents a ribbon produced from a material and under a specific hydraulic pressure).

The tensile strength vs. solid fraction profiles for 81 materials are plotted in Figure 6. The target region (i.e., 0.6 ≤ *SF* ≤ 0.8 and *TS* ≥ 1 MPa) is highlighted by the red color. Each point represents a ribbon produced from a material and under a specific hydraulic pressure. Ribbons form the same material are lined up, and an overall upward trend can be observed. Three categories of materials can be clearly discriminated in accordance with the predefined target. For Category Ⅰ materials, there is at least one point that is located in the target region. By contrast, there is no point that is in the target region for Category Ⅱ and Ⅲ materials. Category Ⅱ materials are characterized by that they can be compacted into ribbons with both TS higher than 1 MPa and SF larger than 0.8. Whereas, the tensile strength for a Category Ⅲ material is less than 1 MPa over the entire pressure range (i.e., 30–110 bar). The classification criteria for each category, as well as the numbers of materials belonging to each category, are shown in Table 2.

As can be seen from Table 2, a total of 29 materials, including 24 pharmaceutical excipients and 5 NPPs, are classified into Category Ⅰ. Moreover, two subcategories (named as Category ⅠA and Category ⅠB) can be distinguished from Category Ⅰ. When compacted, ribbons from Category ⅠA powders can reach the target region at the low hydraulic pressure range of 30–70 bar. A total of 18 cellulose materials, such as nine batches of MCC (No. 1 to No. 3 and No. 5 to No. 10), five batches of HPMC (No. 19 to No. 23), one batch of MC (No. 11, Viscosity 4000 cps.) and one batch of L-HPC (No. 24), belong to this subcategory. MCC is a widely used excipient in dry granulation because of its excellent compressive properties [19,86,87]. The lactose Cellactose^®^ 80 also belongs to this subcategory since it contains 25% cellulose. Besides, one batch of DCP (No. 42), one batch of CCNa (No. 45), two batches of PVPP (No. 48 and 49) and 5 NPPs are included in Category ⅠA. It should be noted that there is risk for Category ⅠA materials that the solid fraction is over 0.8 when the applied pressure is increased. For Category ⅠB materials, ribbons can only reach the target region at the relatively high hydraulic pressure range of 90–110 bar. These materials consist of one batch of DCP (No. 41), one batch of CCNa (No. 46) and one batch of CMC-Ca (No. 50).

A total of 41 batches of powders are classified as Category Ⅱ materials, including 21 pharmaceutical excipients and 20 NPPs. Most of NPPs are belonging to this group. According to the ribbon performance under different hydraulic pressures, Category Ⅱ materials can be further divided into Category ⅡA and Category ⅡB. The tensile strength of a ribbon compacted from the Category ⅡA material can exceed 1 MPa at the relatively low-pressure range of 30–70 bar. While, Category ⅡB materials can only be compacted into ribbons with tensile strength larger than 1 MPa at the relatively high-pressure range of 90–110 bar. Category ⅡA materials contain one batch of MCC (No. 4), one batch of MC (No. 12, Viscosity 15 cps.), five batches of EC (No. 13 to No. 17), one batch of HPMC (No. 18), two batches of lactose (No. 28 and No. 31), mannitol, two batches of D-Sorbitol (No. 39 and 40), sodium bicarbonate, malic acid and 12 NPPs. Category ⅡB materials consist of two batches of lactose (i.e., No. 26 and No. 29), corn starch, pregelatinized starch, dextrin, D-glucose and eight NPPs.

The remaining 11 batches of materials in the material library are classified as Category Ⅲ materials, which contain eight pharmaceutical excipients and three NPPs. Category Ⅲ materials are characterized by unfavorable tensile strength performance. Excipients include three batches of lactose (i.e., Duralac^®^ H, Tablettose^®^ 80 and Pharmatose^®^ 200M), sucrose (No. 40), two batches of CMS-Na (No. 43 and 44), one batch of CCNa (No. 47) and calcium phosphate (No. 51) are included in this group. The porosity of the ribbons produced by calcium phosphate (No. 51) are 0.5785 and 0.5748 at 90 bar and 110 bar, respectively. It is expected that the porosity of highly porous raw materials remains to a certain extent and increases the porosity of the ribbon. However, as a clear distinction between intra- and inter-particular pores in the ribbons was not possible, it could be conjectured [88]. Two NPPs (No. 71 and 72) that cannot form ribbons and one NPP (No. 67) that forms very weak ribbons are also included in this category.

The transverse splitting is reported to have an adverse effect on the performance of the RC process, since the ribbon may adhere to the roller surface [74]. For Category Ⅰ materials, a total of 141 tangible ribbon samples were prepared, and only 29 ribbons displayed the transversal splitting. The rate of occurrence is 20.3%. By contrast, the occurrence rates of transverse splitting are 54.6% and 55.8% for Category Ⅱ materials and Category Ⅲ materials, respectively. These results indicate that Category Ⅰ materials have favorable RC performance with less occurrence of sticking. On the other hand, the compaction behavior of 82.8% of Category Ⅰ materials can be described by both the R–D model and the power model with R^2^ > 0.7. The values of this ratio for Category Ⅱ materials and Category Ⅲ materials are 73.2% and 45.5%, respectively. This proves that the compaction behavior of Category Ⅰ materials presents a more regular change pattern.

### 3.5. Latent Variable Modeling in Prediction of Ribbon Properties

Latent variable modelling (LVM) methods are extensively used in pharmaceutical development to uncover the multidimensional relationships and interactions among raw material properties, process parameters and product quality attribute [36,41,42,87,89,90,91,92,93]. In this paper, the PLS methods are applied to investigate the influence of raw material physical characteristics, roll compaction descriptors and hydraulic pressures on ribbon critical quality attributes (CQAs), i.e., solid fraction and tensile strength. In Section 3.3, it can be seen that the roll compaction descriptors estimated for some materials are unreliable or unavailable. Therefore, a reduced material database consisting of 58 batches of materials is established. In PLS regression, the qualitative material factor is replaced by 26 quantitative attributes (i.e., 22 physical material properties and four roll compaction descriptors). The hydraulic pressure acts as the critical process parameter. Each material compacted at every level of hydraulic pressure is treated as one observation. Thus, a total of 290 observations (i.e., 58 × 5 = 290) are obtained. Table 3 shows the input and output variables used in the PLS model.

**Figure 7:**
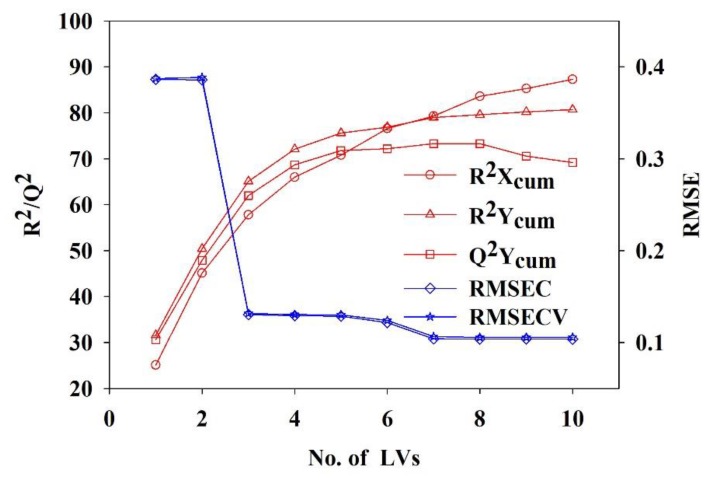
Optimization of the number of latent variables.

Different combinations of input variables, which are expected to have different predictive capabilities for ribbon properties, were investigated. In PLS Model 1, 22 material properties and the hydraulic pressure are combined as predictors. The input variables of PLS Model 2 were four compaction descriptors and the hydraulic pressure. The overall effects of material properties, compaction descriptors and the hydraulic pressure on ribbon properties were investigated in PLS Model 3. The sizes of the calibration dataset for PLS Model 1, Model 2 and Model 3 were 290 × 23, 290 × 5 and 290 × 27, respectively. The two response variables, i.e., the ribbon TS and SF, were predicted simultaneously from one set of latent features. The regression results for three models are shown in Table 4. The number of latent variables (LVs) is optimized according to the trend of the model validation statistics, such as root mean square standard error of calibration (RMSEC), root mean square standard error of cross validation (RMSECV), the fraction of the variation of the X variables explained by the model (R^2^X), the fraction of the variation of the Y variables explained by the model (R^2^Y) and the fraction of the variation of the Y variables predicted by the model (Q^2^Y). Taking Model 3 for example, the chemometric indicators stabilize at 5 LVs that are used to build the model. Increasing the latent variables does not improve the predictive performance of the model, as shown in Figure 7. The cumulative values of R^2^Y (R^2^Ycum) and Q^2^Y (Q^2^Ycum) of PLS Model 3 are 75.6% and 71.8% respectively, which are the highest among the three models. It proves that PLS Model 3 has the best performance in prediction of ribbon properties.

**Figure 8:**
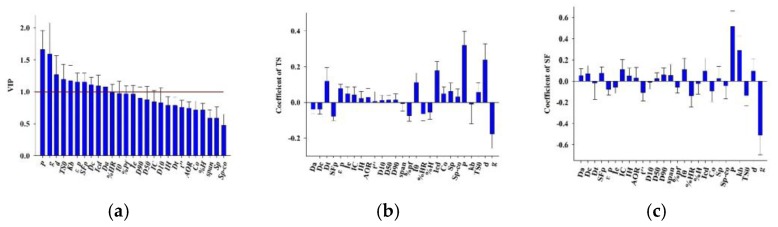
The variable importance in projection (VIP) and the coefficient value of PLS. (**a**) The VIP value of PLS; (**b**) the coefficient plot of tensile strength (TS) values for PLS; and (**c**) the coefficient plot of solid fraction (SF) value for PLS.

Based on the PLS Model 3, the relative importance of independent variables on the overall model are evaluated by the variable importance in projection (VIP) index. A rule of thumb is that variables with VIP > 1 are important. As shown in Figure 8a, the hydraulic pressure has the largest VIP value (VIP = 1.662). This implies that the hydraulic pressure contributes most in explaining the variation in both the X and Y space. Immediately after the hydraulic pressure in the VIP plot are four compaction descriptors including *g*, *d*, *TS*_0_ and *k_b_*, whose VIP values are 1.589, 1.266, 1.193 and 1.170, respectively. The raw material properties including four density related parameters (i.e., *SF_p_*, *ε_p_*, *Dc* and *Da*) and the cohesion index (*Icd*) also have VIP values greater than 1, and these properties are identified as critical raw material attributes (CMAs). The above VIP analysis proves that the ribbon properties are the result of a complex interaction between the hydraulic pressure, the compaction descriptors and material properties, which is consistent with the literature [94]. The regression coefficient plots created for TS and SF are shown in Figure 8b,c, respectively. Variables that have large coefficients are important in the model. It can be seen that the hydraulic pressure is strongly positively correlated with either TS or SF, which is the same as above findings. The compaction descriptor *g* is strongly negatively correlated with TS or SF. Other compaction descriptors attribute different effects to response variables. The cohesion index *Icd* and the compaction descriptors *d* show the positive influence on TS, but they have little influence on SF. The compaction descriptor *k_b_* shows the positive influence on SF, but it has almost none influence on TS.

**Figure 9:**
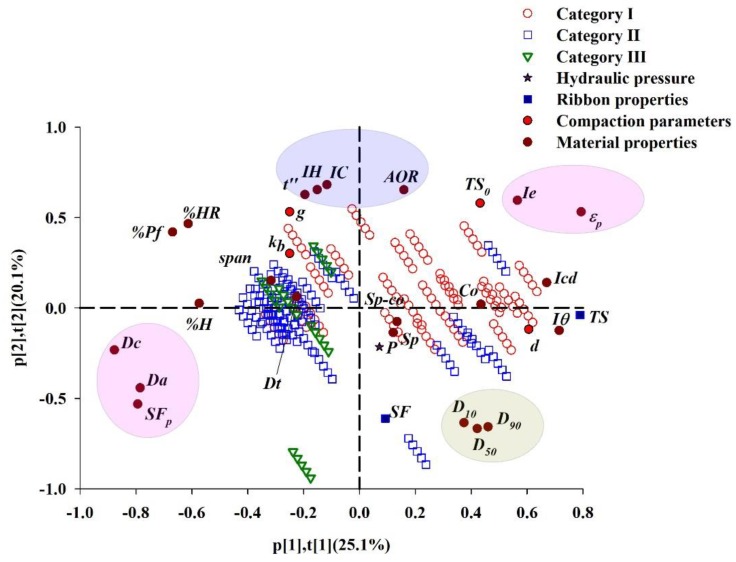
The loading bi-plot for LV-1 vs. LV-2.

In the PLS Model 3, the first and the second LVs fit 25.1% and 20.1% of variability in the dataset, respectively. The Loading bi-plot of the first two LVs is shown in Figure 9, where the loadings and scores are super-imposed for easier interpretation of the relationship between the variables and observations. The loadings are the model variables that explain the relationship between the X and Y variables and the latent variables. Variables far from the center are important for explaining the variation in the dataset. It could be found that TS is mainly explained by the first LV and SF is mainly explained by the second LV. TS is situated near *Icd*, *d* and *Iθ*, indicating that TS is positively correlated with the three variables. While, the density properties of raw materials (i.e., *Da*, *Dc* and *SF_p_*) lie on opposite side of TS, revealing that TS is negatively correlated with *Da*, *Dc* and *SF_p_*. Variables *Da*, *Dc* and *SF_p_* are highly correlated, as are variables *Ie* and *ε_p_*. Material porosity is a measure that describes the amount of void space in the powder bed. This metric, thus is negatively correlated with *SF_p_*, meaning that as the material is less porous, its *SF_p_* is higher [95]. The moisture content (%HR) is on the opposite side of the TS, which conforms the fact that the TS increases as the moisture content decreases [96]. SF is highly correlated with the variable *D*_10_, *D*_50_, *D*_90_. While the compaction descriptor *g* and flow properties of raw materials (i.e., *IH*, *IC*, *t″*, *AOR*,) are situated on its opposite side. The poor flow properties of the powder would result in the production of less uniform ribbons with reduced density [13,32]. The variables *g*, *k_b_* and *P* are close to the center, indicating that they are not significant for explaining the variation in the plotted latent variables. Whereas, at the LV3 vs. LV5 loading plot as shown in Figure S1., it can be seen that *k_b_* and *P* are positively corelated with SF and *g* is negatively corelated with *SF*.

The scores are used to find clusters. Groups of observations that fall in the same area of the score plot have similar X variables. It is clearly observed that the five observations from the same material are lined up along with the direction of the hydraulic pressure and the ribbon SF. There are two materials outside the 95% Hotelling T^2^ confidence limit as shown in Figure S2. They are sucrose (No. 37) and sorbitol (No. 39) that have large particle sizes. The values of *D*_50_ are 267 μm and 395 μm for the sucrose and the sorbitol, respectively. The porosities of the two materials are higher than 0.82 under different hydraulic conditions. At high hydraulic pressure range of 90–110 bar, their porosities are greater than 0.91. Materials with different roll compaction behavior are highlighted by different colored icons: red dot (Category Ⅰ), blue square (Category Ⅱ) and green triangle (Category Ⅲ). Category Ⅰ materials are distributed in the upper right, and are close to TS, indicating that these materials have high tensile strength, consistent with the previous classification of materials. Category Ⅱ and Category Ⅲ materials cannot be discriminated in the score space of the first two LVs. These materials are far away from TS, implying that they are not easy to be compacted into ribbons with high tensile strength.

### 3.6. Development of Multi-Objective Design Space

**Figure 10:**
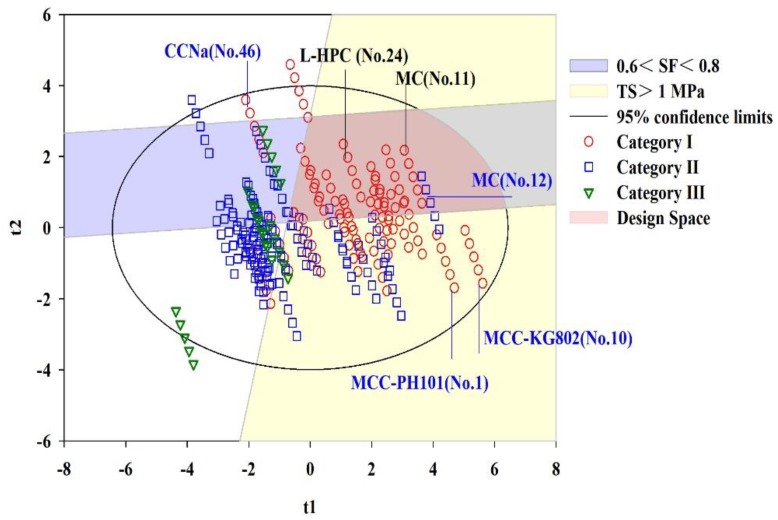
The multi-objective design space developed based on the refined PLS model.

Based on the PLS Model 3, a refined PLS model is developed in order to exclude the effect of noise variables. The new model contains ten variables as inputs whose VIP values are larger than 1 in PLS Model 3. Under four latent variables, the R^2^Xcum, R^2^Ycum and Q^2^Ycum of the refined PLS Model are 79.6%, 73.1% and 71.8% respectively. It suggests that both the calibration and prediction performance of the refined PLS model remains almost the same as the PLS Model 3. The structure of the refined model becomes simpler, and the first and the second LVs can explain 44.3% and 19.1% of variability in the X variables space. The target criteria of ribbons are mapped onto the first two scores’ space of the refined PLS, as shown in Figure 10. The yellow region represents TS ≥ 1 MPa, and the blue region stands for 0.6 ≤ *SF* ≤ 0.8. The circle corresponds to the 95% confidence limit for all observations’ Euclidean distances from the center point in the score space. Under the constraint of the 95% confidence limit, an overlapping area of the yellow region and blue region is highlighted by the red color. The red area represents the multi-objective design space where the multidimensional combinations and interactions between the input materials properties, the hydraulic pressure and the predefined ribbon quality attributes are visualized.

The symbols for materials from different categories are the same as that in Figure 9. It can be observed that most of Category I materials are located within or intersected with the design space. However, seven Category I materials are distributed near but not inside the design space, and four Category Ⅱ materials are intersected with the design space. It may be due to the prediction error of the refined PLS model. For instance, the RMSECV values are 0.05030 and 1.350 MPa for responses SF and TS, respectively. These results demonstrate that the feasible region for RC formulation is narrow. A single powder or powder mixture with similar properties like Category I materials is likely to be projected onto the design space, giving preliminary directions for RC formulation and process design. In future works, more attentions will be paid to RC process-relevant material properties characterization, in order to improve the accuracy of design space.

From both Figure 9 and Figure 10, it is observed that some samples are located near each other, which indicates the redundancy of the material database. Although the data redundancy problem was solved by selection of critical variables as shown above, the selection of representative samples from an existing material library remains to be a challenge. This paper is a part and the beginning of a larger research program. In future studies, the representative materials will be selected under the guidance of the roll compaction behavior classification system (RCBCS) established in this paper, as well as considering the similarities or Euclidean distance between different materials with the help of material properties data or scores from the multivariate calibration model. Then, the interactions between critical raw materials attributes and more critical process parameters will be adequately investigated through design of experiment methodologies under the guidance of QbD and quality risk management (QRM) principles [97]. The freedom of design space built in this paper will be improved to provide more flexibility on adjusting CPPs of the roll compaction process.

## 4. Conclusions

This study has carried out an extensive number of roll compaction experiments using a material library that consists of 81 pharmaceutical materials. The roll compaction behavior classification system (RCBCS) was built and 81 materials were classified into three categories. The multivariate relationships between raw material properties, hydraulic pressures and ribbon quality attributes have been obtained by the PLS algorithm. The density related parameters and the cohesion index were identified as critical raw material attributes. Furthermore, a refined PLS model was built by removing redundant input variables. A multi-objective design space was developed under the latent variable space, summarizing the material characteristics that led to favorable RC performance. The RCBCS presented in this paper enables a formulator to perform the risk assessment of any new materials, and the data modeling method facilitates enhanced understanding of the impact of formulation ingredients on the strength and porosity of compacts.

## Figures and Tables

**Table 1:** The frequency of splitting occurrences under different hydraulic pressures.

Splitting Mode		Splitting Occurrences Under Different Pressures				
		30 bar	50 bar	70 bar	90 bar	110 bar
T	SUM	39	40	34	31	26
L	L2	4	15	20	23	23
	L3	0	0	1	1	0
	LJ	3	8	18	19	22
	LN	3	3	4	5	4
	SUM	10	26	43	48	49

**Table 2:** The roll compaction behavior classification criteria and the number of materials belonging to each category.

Main Category	Main Criteria	Subcategory	Second Criteria	No. of Excipients	No. of NPPs
Ⅰ	0.6 ≤ SF ≤ 0.8 and TS ≥ 1 MPa	ⅠA	Fulfilling the main criteria at 30–70 bar	21	5
		ⅠB	Fulfilling the main criteria at 90–110 bar	3	0
Ⅱ	SF > 0.8 and TS ≥ 1 MPa	ⅡA	Fulfilling the main criteria at 30–70 bar	14	12
		ⅡB	Fulfilling the main criteria at 90–110 bar	7	8
Ⅲ	TS < 1 MPa	/	/	8	3

**Table 3:** Description of input and output variables for the partial least squares (PLS) model.

	Variable Type	
Input	Material property	*D*_10_, *D*_50_,*D*_90_, *span*, *%Pf*, *Iθ*, *Da*, *Dc*, *D_t_*, *SF_p_*, *ε_p_*, *IH*, *IC*, *Ie*, *t″*, *AOR*, *%HR*, *%H*, *Icd*, *Co*, *Sp*, *Sp-co*
	Compaction descriptor	*k_b_*, *TS*_0_, *d*, *g*
	Hydraulic pressure	*P*
Output	Ribbon property	*TS*, *SF*

**Table 4:** Diagnostics of three PLS models with different input variables.

Model	LVs	R^2^Xcum	R^2^Ycum	Q^2^Ycum
1	4	71.9%	54.7%	51.1%
2	4	95.5%	67.5%	64.9%
3	5	70.8%	75.6%	71.8%

## Supplementary Materials

The following are available online at https://www.mdpi.com/1999-4923/11/12/662/s1. Supplementary Materials 1: Table S1: The detailed roll compaction information for each material, including materials names, abbreviations, lot numbers, suppliers, physical properties, compaction descriptors, the ribbon *SF* and *TS* under different hydraulic pressures, the ribbon splitting behavior and the roll compaction. Supplementary Materials 2: Figure S1. The loading plot for LV3 vs. LV5. Figure S2. The score plot with 95% Hotelling T^2^ confidence limit.
